# Impact of cooking oil fume exposure and fume extractor use on lung cancer risk in non-smoking Han Chinese women

**DOI:** 10.1038/s41598-020-63656-7

**Published:** 2020-04-21

**Authors:** Tzu-Yu Chen, Yao-Hwei Fang, Hui-Ling Chen, Chin-Hao Chang, Hsin Huang, Yi-Song Chen, Kuo-Meng Liao, Hsiao-Yu Wu, Gee-Chen Chang, Ying-Huang Tsai, Chih-Liang Wang, Yuh-Min Chen, Ming-Shyan Huang, Wu-Chou Su, Pan-Chyr Yang, Chien-Jen Chen, Chin-Fu Hsiao, Chao A. Hsiung

**Affiliations:** 1 Institute of Population Health Sciences, National Health Research Institutes, Zhunan, Taiwan; 2 Department of Medical Research, National Taiwan University Hospital, Taipei, Taiwan; 3 Department of Nursing, Fu Jen Catholic University, Taipei, Taiwan; 4 Department of Microbiology, National Taiwan University College of Medicine, Taipei, Taiwan; 5 Division of Endocrinology & Metabolism, Taipei City Hospital, Zhongxiao Branch, Taipei, Taiwan; 6 Faculty of Medicine, School of Medicine, National Yang-Ming University, Taipei, Taiwan; 7 Division of Chest Medicine, Department of Internal Medicine, Taichung Veterans General Hospital, Taichung, Taiwan; 8 Department of Pulmonary and Critical Care Medicine, Chiayi Chang Gung Memorial Hospital, Chang Gung Medical Foundation, Chiayi, Taiwan; 9 Department of Thoracic Medicine, Chang Gung Memorial Hospital, Taoyuan, Taiwan; 10 School of Medicine, National Yang-Ming University, Taipei, Taiwan; 11 Department of Chest Medicine, Taipei Veterans General Hospital, Taipei, Taiwan; 12 Department of Internal Medicine, E-Da Cancer Hospital, School of Medicine, I-Shou University and Kaohsiung Medical University, Kaohsiung, Taiwan; 13 Department of Internal Medicine, National Cheng Kung University Hospital, College of Medicine, National Cheng Kung University, Tainan, Taiwan; 14 Department of Internal Medicine, National Taiwan University Hospital, Taipei, Taiwan; 15 Genomics Research Center, Academia Sinica, Taipei, Taiwan

**Keywords:** Risk factors, Cancer

## Abstract

Smoking tobacco is the major risk factor for developing lung cancer. However, most Han Chinese women with lung cancer are nonsmokers. Chinese cooking methods usually generate various carcinogens in fumes that may inevitably be inhaled by those who cook the food, most of whom are female. We investigated the associations of cooking habits and exposure to cooking fumes with lung cancer among non-smoking Han Chinese women. This study was conducted on 1,302 lung cancer cases and 1,302 matched healthy controls in Taiwan during 2002–2010. Two indices, “cooking time-years” and “fume extractor use ratio,” were developed. The former was used to explore the relationship between cumulative exposure to cooking oil fumes and lung cancer; the latter was used to assess the impact of fume extractor use for different ratio-of-use groups. Using logistic models, we found a dose–response association between cooking fume exposure and lung cancer (odds ratios of 1, 1.63, 1.67, 2.14, and 3.17 across increasing levels of cooking time-years). However, long-term use of a fume extractor in cooking can reduce the risk of lung cancer by about 50%. Furthermore, we provide evidence that cooking habits, involving cooking methods and oil use, are associated with risk of lung cancer.

## Introduction

Lung cancer has been the leading cause of cancer death for women worldwide, including in Taiwan^1,2^. The incidence trend of lung cancer has increased steadily in Taiwan^2^. It is well known that cigarette smoking is the major cause of lung cancer^3,4^. However, unlike for Western populations, the epidemiological features of lung cancer in Han Chinese women cannot be elucidated fully by smoking behavior^5,6^, because the majority of Han Chinese female lung cancer cases have been nonsmokers^5,7–12^. In fact, in Taiwan about 9–10% of females with lung cancer and 79–86% of males with lung cancer were tobacco smokers^13–15^. The discrepancy in tobacco usage for female and male lung cancer patients in Taiwan suggests that other potential risk factors linked to lung cancer for non-smoking women might require further investigation.

Previous reports show that about 90% of non-smoking women regularly cook family meals^10^. Given that a common step in Chinese-style cooking involves heating oil until it smokes before adding the food, various carcinogens can be produced and inevitably be inhaled by the cook^6,16–19^. Therefore, exposure to cooking oil fumes (COFs) may be an important risk factor for lung cancer in non-smoking Han Chinese women. There is growing evidence that exposure to COFs is linked to lung cancer^17^, with several studies showing that long-term exposure to COFs may be linked to lung cancer^5,14,16,20–22^. For example, a case-control study was conducted in Taiwan in 1993–1996 to explore the association of oil fumes with lung cancer in women, with 131 non-smoking female lung cancer cases and two sets of controls (hospital and community controls, with sample sizes of 252 and 262, respectively)^14^; this study found that lung cancer risk increased with the number of meals per day to about threefold for women who cooked these meals each day. Another case-control study of lung cancer conducted in Montreal among 466 female lung cancer cases and 616 female healthy controls found an odds ratio (OR) of 2.5 (95% confidence interval (CI): 1.5, 3.6) for women exposed to traditional heating and cooking sources^23^. Furthermore, a population-based case-control study in Hong Kong showed that cumulative exposure to cooking through any forms of frying could increase the risk of lung cancer among non-smoking women^24^; the same study also found that participants who used a fume extractor or an exhaust fan did not have a significantly lower risk of lung cancer (OR = 0.94, 95% CI: 0.43, 2.02). However, two recent meta-analyses both demonstrated the significantly protective effect of fume extractor use and good ventilation on lung cancer risk, one of which further concluded that cooking methods may have different impacts on lung cancer risk^25,26^.

Most previous studies on this subject were conducted with small to moderate sample sizes. Even though the recent meta-analyses were based on a collection of studies with larger sample sizes, they evaluated the length and intensity (frequency) of COF exposure separately only or did not adequately take into account the effect of various potential confounding factors. Therefore, we conducted a case-control study with a large sample size among non-smoking Han Chinese women to investigate the association between COFs and lung cancer risk by using a composite index that takes both lifetime cooking duration and cooking times into account. The composite index facilitates measurement of the cumulative exposure to COFs, similar to the one used in the Hong Kong study^24^, and was used in our earlier publication^10^.

In addition, previous studies on this topic focused mainly on the impact of good and poor kitchen ventilation while cooking, whereas we considered how long a fume extractor was used during participants’ lifetime cooking years and assessed the association between use of fume extractors and lung cancer risk. The associations of different cooking methods (pan-frying, stir-frying, and deep-frying) and different types of cooking oils (lard and vegetable oil) on lung cancer were also investigated in this study. All these analyses were adjusted for more potential confounders, including age, education level, lung cancer in first-degree relatives, second-hand smoke (SHS), history of hormone-replacement therapy, history of oral contraceptive use, and person’s role as homemaker and/or chef.

## Materials and Methods

### Study population

This is a case-control study, a sub-study of the Genetic Epidemiological Study of Lung Adenocarcinoma (GELAC) in Taiwan. Although the purpose of the GELAC study was to identify genetic determinants that influence susceptibility to lung adenocarcinoma, we collected all histological lung cancer types to compare the clinical and etiological differences in lung adenocarcinoma and other lung carcinomas. Details of the GELAC study have been described previously^27–30^.

Briefly, all lung cancer cases and healthy controls were recruited from six tertiary medical centers in Taiwan: National Taiwan University Hospital, Taipei Veterans General Hospital, Chang Gung Memorial Hospital, Taichung Veterans General Hospital, National Cheng Kung University Hospital, and Kaohsiung Medical University Hospital. All histologically or cytologically confirmed lung cancer cases aged 18 years or older (with no upper age limit) were recruited from 2002 to 2010. Those with metastatic lung cancer, a prior history of other cancer, or lack of suitable blood specimen were excluded. The controls were randomly enrolled from people who received periodic comprehensive physical examinations at the health examination departments in any of the six hospitals during the study period. They were cancer-free and selected by matching with cases on five-year age group, sex, smoking status, and education level. Those who lacked a suitable blood specimen or had a prior history of cancer, chronic bronchitis, or emphysema were excluded. In the present study, only non-smoking female lung cancer cases and non-smoking female controls were included in the analysis. The study had a total of 1,302 non-smoking female lung cancer cases and 1,302 matched controls, where 84.6% of the cases were the lung adenocarcinoma subtype. This study received approval from the institutional review board of each hospital and of the National Health Research Institutes, Taiwan; and all participants provided written informed consent. All methods were carried out in accordance with the approved guidelines and regulations.

### Data collection

Standardized interview-administered questionnaires were used to collect information on basic demographics, the ages at which the participant started and ended cooking, habit of daily cooking times, frequency of cooking methods, types of cooking oils used, fume extractor usage, family history of lung cancer, SHS exposure, history of hormone-replacement therapy, history of oral contraceptive use, homemaker status, and chef status. Once the consent forms were obtained, personal interviews with all participants were conducted by trained interviewers. Of them, 8.1% were proxy interviews. For the lung cancer cases, the mean time between diagnosis and interview was 0.6 years, with a standard deviation of 1.45. On-site training and ongoing monitoring were also performed to ensure standardized data collection and to avoid discrepancies in data-collection practices between the six hospitals. From data collection to entering the data into the database, each step followed a well-established standard operating procedure. The database was established through a quality-control and double-entry process at the National Health Research Institutes in Taiwan.

In this study, a nonsmoker was defined as (1) a subject who has never smoked, or (2) a subject who has not smoked regularly (defined as smoking at least one cigarette per week) for six months at any given period of her lifetime^27,31^. Three types of SHS exposure were defined: a history of inhaling other people’s cigarette smoke in the workplace, a history of living with family members who smoked, and regular exposure to SHS from friends or relatives. Lung cancer family history included lung cancer histories of first-degree relatives (i.e., parents, brothers, sisters, and biological children). People were categorized as receiving hormone-replacement therapy/oral contraceptive treatment if this treatment had lasted at least three months. People were categorized as homemakers if they spent more than half of their lifetime being a full-time homemaker. People were categorized as chefs if they had ever been a chef.

For each participant who had cooked at least six months continuously, we evaluated her cumulative exposure to COFs through an index of cooking time-years, defined as follows: cooking time-years = $\sum _{r}{d}_{r}\times {y}_{r}$, where ${d}_{r}$ = average daily times of cooking in age range *r*, and ${y}_{r}$ = years of cooking in age range *r*. For example, a woman who cooked three meals per day for the 20 consecutive years between the ages of 21 and 40 would have cooking time-years of 60 for that age range. In our questionnaire, each participant was asked the ages at which she started and ended cooking, and the daily cooking times while she cooked in the three age ranges (0–20, 21–40, and ≥41 years old). The cooking time-years index is computed by summing the cooking time-years in the three age ranges. For any participant who had not cooked at least six consecutive months, we defined her cooking time-years to be zero.

Another new variable, “fume extractor use ratio,” was developed to represent the proportion of fume extractor use over the total lifetime of cooking time: fume extractor use ratio = (total number of years of fume extractor use)/(total number of cooking years).

For a participant, we counted the number of cooking days weekly for different cooking methods (pan-frying, stir-frying, and deep-frying). In our questionnaire, each participant was asked the number of cooking days per week using each method in the three age ranges (0–20, 21–40, and ≥41 years old). We used the cooking time-years in each age range divided by the total cooking time-years as a weight, and then calculated the weighted average of cooking days weekly for the three individual cooking methods.

The participants had available information concerning which types of cooking oils were frequently used in cooking, and we categorized them into three groups: frequent use of vegetable oil, frequent use of lard, and use of both.

### Statistical analysis

To maximize the sample size for the statistical analysis, we broke the case-control matching and used unconditional logistic regression models with the adjustment for the original matching variables^32^. This is because, when a participant (either case or control) had missing covariates, a matched pair would be eliminated from the 1:1 matched-samples analysis. In this study, associations were analyzed using the glm function in R 3.4.3 software.

To explore the association between COF exposure and lung cancer, the composite index of cooking time-years was categorized into five levels (≤10, 11–60, 61–110, 111–160, and >160), with the lowest one serving as reference (no exposure to COFs). ORs with a 95% CI for lung cancer related to cooking time-years were estimated, adjusting for age (continuous), education (illiterate, elementary school, junior high school, senior/vocational high school, college, graduate school), lung cancer in first-degree relatives (yes/no), SHS at home (yes/no) and the workplace (yes/no), SHS of relatives who smoked (yes/no), history of hormone-replacement therapy (yes/no), history of oral contraceptive use (yes/no), homemaker (yes/no), and chef (yes/no). The dose–response association between lung cancer and different levels of exposure to COFs was further estimated by trend test. To estimate the population attribution risk (PAR) due to the COF exposure for lung cancer, all participants were grouped into two categories based on their cooking time-years: exposure to COF (cooking time-years >10) and no COF exposure (cooking time-years ≤10). The PAR was calculated based on the exposure rate in controls and the adjusted OR from the logistic regression model.

To further study the association between use of a fume extractor and lung cancer, the fume extractor use ratio was grouped into three levels: 0 ≤ ratio <0.33 for short-term use, 0.33 ≤ ratio <0.67 for medium-term use, and 0.67 ≤ ratio ≤ 1 for long-term use. Only participants who were exposed to COFs (cooking time-years >10) were included in the analysis. ORs with a 95% CI for lung cancer related to the fume extractor use ratio were estimated, adjusting for age, education, categories of cooking time-years (11–60, 61–110, 111–160, and >160), lung cancer in first-degree relatives, SHS at home and the workplace, SHS of relatives who smoked, history of hormone-replacement therapy, history of oral contraceptive use, homemaker status, and chef status.

Different cooking methods and different types of cooking oils were also investigated for understanding the association between cooking habits and lung cancer risk. For the habit of using two types of cooking oil, all the participants were grouped into three categories: frequent use of vegetable oil, frequent use of lard, and use of both. The first category was used as a reference. For the habit of each cooking method, all the participants were grouped into two categories based on their frequencies: high level if her cooking days weekly of the method were larger than the median; low level otherwise. ORs with a 95% CI for the habit of using cooking oils and for frequency of the three methods were separately estimated, adjusting for the same covariates as above.

## Results

Table 1 shows the basic demographic characteristics of the 1,302 lung cancer cases and the 1,302 matched healthy controls. The demographic parameters history of hormone-replacement therapy, history of oral contraceptive use, and history of serving as a chef, as well as the matched age, had similar distributions across case and control samples; and the matched education levels were equally distributed between the two groups. Cases had more exposure to COFs, more lung cancer in first-degree relatives, and more SHS exposure than controls; and more controls than cases had ever been homemakers. Table 2 summarizes the cooking habits among cases and controls; in terms of extractor use, fewer cases than controls used a fume extractor in the kitchen (94.9% vs. 97.8%, respectively). More controls than cases had long-term use of a fume extractor (54.6% vs. 51.5%, respectively). The cases who used the pan-frying method frequently were more common than the controls (50.9% vs. 40.6%, respectively), and more cases than controls used lard frequently (4.9% vs. 2.6%, respectively).

**Table 1:** Distributions of demographic characteristics among 1,302 lung cancer cases and 1,302 matched healthy controls in the Taiwan GELAC study during 2002–2010.

Variables	Controls	Cases
	N (%)	N (%)
Age		
Mean (years)	58.62	58.55
Education		
Illiterate	197 (18.1)	197 (18.1)
Elementary school	425 (39.1)	425 (39.1)
Junior high school	180 (16.6)	180 (16.6)
Senior/Vocational high school	256 (23.6)	256 (23.6)
College	224 (20.6)	224 (20.6)
Graduate school	20 (1.8)	20 (1.8)
Lung cancer in first-degree relatives		
Yes	49 (3.8)	135 (10.4)
No	866 (66.5)	1107 (85)
Unknown	387 (29.7)	60 (4.6)
Second-hand smoking: Home		
Yes	740 (56.8)	851 (65.4)
No	528 (40.6)	425 (32.6)
Unknown	34 (2.6)	26 (2)
Second-hand smoking: Work		
Yes	200 (15.4)	259 (19.8)
No	1102 (84.6)	1041 (80)
Unknown	0 (0)	2 (0.2)
Second-hand smoking: Relatives		
Yes	286 (21.9)	333 (25.6)
No	1015 (78)	966 (74.2)
Unknown	1 (0.1)	3 (0.2)
History of hormone-replacement therapy		
Yes	239 (18.4)	230 (17.7)
No	982 (75.4)	1018 (78.2)
Unknown	81 (6.2)	54 (4.1)
History of oral contraceptive use		
Yes	95 (7.3)	113 (8.7)
No	1142 (87.7)	1116 (85.7)
Unknown	65 (5)	73 (5.6)
Homemaker		
Yes	136 (10.4)	91(7.0)
No	1,147 (88.1)	1,206 (92.6)
Unknown	19 (1.5)	5 (0.4)
Chef		
Yes	82 (6.3)	84 (6.2)
No	1,220 (93.7)	1,217 (93.7)
Unknown	0 (0)	1 (0.1)
Exposure to COFs (cooking time-years >10)		
Yes	1069 (82.1)	1135 (87.2)
No	204 (15.7)	153 (11.8)
Unknown	29 (2.2)	14 (1.1)

Abbreviations: COF, cooking oil fume.

**Table 2:** Comparisons of cooking habits between cases and controls who had ever cooked, from the Taiwan GELAC study during 2002–2010.

Variables	Controls	Cases
	N = 1069	N = 1135
Age began cooking, N (%)		
<20	287 (26.8)	318 (28.0)
20–24	483 (45.2)	483 (42.6)
≥25	299 (28.0)	334 (29.4)
Fume extractor in kitchen, N (%)		
Yes	1045 (97.8)	1077 (94.9)
No	24 (2.2)	58 (5.1)
Fume extractor use ratio, N (%)		
Short-term use (0 ≤ ratio <0.33)	75 (7.0)	123 (10.8)
Medium-term use (0.33 ≤ ratio <0.67)	243 (22.7)	266 (23.4)
Long-term use (0.67 ≤ ratio ≤ 1)	584 (54.6)	584 (51.5)
Unknown	167 (15.6)	162 (14.3)
Cooking oils, N (%)		
Frequent use of vegetable oil	525 (49.1)	548 (48.3)
Frequent use of lard	28 (2.6)	56 (4.9)
Both used	515 (48.2)	527 (46.4)
Unknown	1 (0.1)	4 (0.4)
Pan-frying^a^		
CDW ≤ 5	578 (54.1)	436 (38.4)
CDW > 5	434 (40.6)	578 (50.9)
Unknown, N (%)	57 (5.3)	121 (10.7)
Stir-frying^a^		
CDW < 7	303 (28.3)	272 (24.0)
CDW = 7	753 (70.4)	760 (67.0)
Unknown, N (%)	13 (1.3)	103 (9.0)
Deep-frying^a^		
CDW = 0	622 (58.2)	570 (50.2)
CDW > 0	426 (39.9)	461 (40.6)
Unknown, N (%)	21 (2.0)	104 (9.2)

Abbreviations: CDW, weighted average of cooking days weekly.

^a^The medians of CDW for pan-frying, stir-frying, and deep-frying are 5, 7, and 0, respectively.

Table 3 shows that, without any adjustments, the highest level of cooking time-years (>160) was significantly associated with an increased risk of lung cancer, with a crude OR of 1.9 (95% CI: 1.24, 2.93). To evaluate the effect of cooking time-years on lung cancer risk, a multivariate model was performed to adjust potential confounders. Here we discarded those data with missing values, so we used only 1,109 cases and 787 controls for our analysis. The adjusted ORs of lung cancer risk across increasing levels of cooking time-years (11–60, 61–110, 110–160, and >160) were 1.63 (95% CI: 1.20, 2.23), 1.67 (95% CI: 1.12, 2.49), 2.14 (95% CI: 1.19, 3.90), and 3.17 (95% CI: 1.34, 7.68), respectively, relative to cooking time-years ≤10. They present an increased dose–response trend with *P*_trend_ = 0.0297. The PAR% due to the COF exposure for lung cancer is 7.83%. We also compared the risks of these groups to the group having no COF exposure, adjusting for the ten covariates in Table 3 and, additionally, categories of fume extractor use ratio (short-term use, medium-term use, and long-term use). A similar result was observed: the adjusted ORs were 1.64 (95% CI: 1.11, 2.44), 1.59 (95% CI: 1.01, 2.52), 2.39 (95% CI: 1.23, 4.68), and 3.14 (95% CI: 1.20, 8.41).

**Table 3:** Odds ratios and 95% confidence intervals for cooking time-years and risk of lung cancer between cases and controls in the Taiwan GELAC study during 2002–2010.

Cooking time-years	Crude model				Adjusted model			
	Controls (1273)		Cases (1288)		Controls (787)		Cases (1109)	
	N (%)	N (%)	OR	95% CI	N (%)	N (%)	aOR^a^	95% CI
≤10	204 (16.0)	153 (11.9)	1		148 (18.8)	143 (12.9)	1	
11–60	378 (29.7)	434 (33.7)	1.53	1.19, 1.97^*b^	239 (30.4)	374 (33.7)	1.63	1.20, 2.23^*^
61–110	421 (33.1)	395 (30.7)	1.25	0.97, 1.61	252 (32.0)	338 (30.5)	1.67	1.12, 2.49^*^
111–160	223 (17.5)	239 (18.6)	1.43	1.08, 1.89^*^	121 (15.4)	197 (17.8)	2.14	1.19, 3.90^*^
>160	47 (3.7)	67 (5.2)	1.9	1.24, 2.93^*^	27 (3.4)	57 (5.1)	3.17	1.34, 7.68^*^
*P*_trend_ = 0.0297								

Abbreviations: aOR, adjusted odds ratio; OR, odds ratio; CI, confidence interval.

^a^aOR: The OR was adjusted for age, education, lung cancer in first-degree relatives, exposure to second-hand smoke at home and the workplace, exposure to second-hand smoke of relatives who smoked, history of hormone-replacement therapy, history of oral contraceptive use, homemaker status, and history of being a chef.

^b^*Indicates that the 95% CI of the OR does not include 1.

Table 4 displays the associations between fume extractor use ratio and lung cancer risk. Long-term use (0.67 ≤ ratio ≤ 1) and medium-term use (0.33 ≤ ratio <0.67) of the fume extractor were significantly associated with a decreased risk of lung cancer, with adjusted ORs of 0.49 (95% CI: 0.32, 0.76) and 0.66 (95% CI: 0.42, 1.01), respectively. Long-term use of the fume extractor was more effective than medium use in reducing risk of lung cancer. Stratification analyses demonstrated the consistency of the association results that use of a fume extractor may reduce risk of lung cancer in all subset analyses stratified by cooking time-years. In the low cooking time-years exposure group (11–110), long-term use of the fume extractor during participants’ lifetime cooking years significantly decreased the risk of lung cancer, for which the adjusted OR was 0.56 (95% CI: 0.33, 0.94). A more pronounced reduction was observed in the high cooking time-years exposure group (>110), which had an adjusted OR of 0.37 (95% CI: 0.16, 0.83). The comparisons of the risks of the two groups (those with long-term use of fume extractor under the different categories of exposure, apart from the reference category (≤10)) to the group having no (≤10) exposures to COFs (without regard to their fume extractor use habits) were further presented in Supplementary Table 1.

**Table 4:** Adjusted odds ratios and 95% confidence intervals of fume extractor use ratio between lung cancer cases and healthy controls in the Taiwan GELAC study during 2002–2010.

	Controls	Cases	aOR^a^	95% CI
	N (%)	N (%)		
**Overall (Cooking time-years** > **10)**				
Short-term use (ratio 0–0.33)	41 (7.3)	107 (12.7)	1	
Medium-term use (ratio 0.34–0.66)	144 (25.8)	226 (26.8)	0.66	0.42, 1.01
Long-term use (ratio 0.67–1)	374 (66.9)	510 (60.5)	0.49	0.32, 0.76*^b^
**Cooking time-years 11–110**				
Short-term use (ratio 0–0.33)	26 (5.9)	59 (9.2)	1	
Medium-term use (ratio 0.34–0.66)	80 (18.2)	128 (20.1)	0.75	0.42, 1.33
Long-term use (ratio 0.67–1)	333 (75.9)	451 (70.7)	0.56	0.33, 0.94*
**Cooking time-years** > **110**				
Short-term use (ratio 0–0.33)	15 (12.5)	48 (23.4)	1	
Medium-term use (ratio 0.34–0.66)	64 (53.3)	98 (47.8)	0.58	0.28, 1.15
Long-term use (ratio 0.67–1)	41 (34.2)	59 (28.8)	0.37	0.16, 0.83*

Abbreviations: aOR, adjusted odds ratio; OR, odds ratio; CI, confidence interval.

^a^aOR: The OR was adjusted for age, education, categories of cooking time-years, lung cancer in first-degree relatives, exposure to second-hand smoke at home and the workplace, exposure to second-hand smoke of relatives who smoked, history of hormone-replacement therapy, history of oral contraceptive use, homemaker status, and history of being a chef.

^b^*indicates that the 95% CI of the OR does not include 1.

Table 5 displays the associations between cooking habits and lung cancer. With regard to cooking methods, frequent use of the pan-frying method was significantly associated with an increased risk of lung cancer, with an adjusted OR of 1.53 (95% CI: 1.23, 1.89). There was no significant association for the stir-frying or deep-frying methods. With regard to cooking oils, frequent use of lard was significantly associated with an increased risk of lung cancer, with an adjusted OR of 1.92 (95% CI: 1.04, 3.75), relative to frequent use of vegetable oil.

**Table 5:** Odds ratios and 95% confidence intervals for different cooking habits and risk of lung cancer between cases and controls in the Taiwan GELAC study during 2002–2010.

Cooking habits		Controls	Cases		
		N (%)	N (%)	aOR^b^	95% CI
**Cooking methods**^a^					
Pan-frying	Low frequency (CDW ≤ 5)	383 (57.8)	415 (45.0)	1	
	High frequency (CDW > 5)	280 (42.2)	507 (55.0)	1.53	1.23, 1.89*^c^
Stir-frying	Low frequency (CDW < 7)	224 (32.7)	270 (28.8)	1	
	High frequency CDW = 7)	462 (67.3)	667 (71.2)	1.10	0.87, 1.40
Deep-frying	Low frequency (CDW = 0)	411(60.1)	523 (54.9)	1	
	High frequency (CDW > 0)	273 (39.9)	411 (45.1)	1.13	0.92, 1.41
**Cooking oils**					
Vegetable oil frequently	374 (54.1)	522 (50.8)	1		
Vegetable oil and lard	305 (44.1)	461 (44.8)	0.99	0.79, 1.24	
Lard frequently	15 (1.7)	45 (4.4)	1.92	1.04, 3.75*	

Abbreviations: aOR, adjusted odds ratio; CDW, weighted average of cooking days weekly; CI, confidence interval.

^a^The medians of CDW for pan-frying, stir-frying, and deep-frying are 5, 7, and 0, respectively.

^b^aOR: The OR was adjusted for age, education, categories of cooking time-years, lung cancer in first-degree relatives, exposure to second-hand smoke at home and the workplace, exposure to second-hand smoke of relatives who smoked, history of hormone-replacement therapy, history of oral contraceptive use, homemaker status, and history of being a chef.

^c^*indicates that the 95% CI of the OR does not include 1.

## Discussion

In this study, we used a composite index, “cooking time-years,” to measure the magnitude of exposure to COFs, combining both cooking frequency (cooking times per day) and duration (years). In addition, we developed the “fume extractor use ratio” index, which considers not only the duration of fume extractor use but also total lifetime cooking time. Our study presents evidence that, among non-smoking Han Chinese women, exposure to COFs increases the risk of developing lung cancer, but that using a fume extractor while cooking can reduce the risk. Our results are consistent with previous case-control studies^5,10,14^ and meta-analysis studies^25,26^. Because the sample size in this study is fairly large and more potential confounders are included for adjustment in our analyses, our results may be more valid and detailed than earlier work.

Cooking time-years was used as a surrogate for COFs in this research. Compared with non-smoking female adults who cooked for 10 or fewer cooking time-years, the adjusted ORs in the 110–160 and >160 categories were higher than 2 for lung cancer (Table 3). Our data further demonstrate a dose–response association between cumulative exposure to COFs and lung cancer, after adjusting for the potential confounders. Although some previous reports^16,21^ showed various results of cooking duration to lung cancer risk and the effects of number of meals cooked daily to lung cancer risk varied in different studies as well^14,33^, in the present study we used a composite measurement to combine both cooking intensity and length; and we successfully demonstrated a clear dose–response trend of exposure to COFs, consistent with a study conducted in Hong Kong using a similar measurement system^24^. Thus, we propose that “cooking time-years” be used in future research to quantify COF exposure, which would allow comparable results among various studies.

The “fume extractor use ratio” index facilitates the assessment of the impact for different ratio-of-use groups of a fume extractor. The meta-analysis (2,641 cases and 4,076 controls) of Xue and colleagues^25^ demonstrated that exposure to COFs significantly increased the risk of lung cancer in Chinese non-smoking women (OR = 1.74; 10 studies), especially among those not using a fume extractor (OR = 2.11; 4 studies). Another meta-analysis of Jia and colleagues^26^ that included 23 observational studies also showed that poor ventilation had a pooled OR of 1.2 compared to good ventilation. Our findings not only support the result that the use of a fume extractor shows a significant protective effect against lung cancer but further indicate that long-term use could reduce the risk of lung cancer by more than 50% among non-smoking women, and in particular more than 60% for those who cook more often (Table 4, Supplementary Table 1).

Indeed, in the past few decades in Taiwan, cooking range hoods have become common for fume extraction in the kitchen. They typically are installed 60–80 cm above the stove and therefore generally perform better at capturing cooking fumes and venting them outside than, for example, an exhaust fan fitted in a kitchen window. In view of this, we think that the “fume extractor use ratio” index in our study is well representative of the level of reducing cooking fume exposure due to use of a fume extractor.

COFs appear to be a strong risk factor for lung cancer and chronic bronchitis^19^. Some studies also indicate that components of COFs cause inflammation, apoptosis, cell damage in A549 cells, oxidative DNA damage, DNA adduct formation, and lung carcinogenesis^34–38^. Based on our results we suggest that use of a fume extractor should be regarded as essential to reduce lung cancer risk and other health hazards.

Different cooking habit distributions were observed between cases and controls in this study. More cases frequently used the “pan-frying” cooking method than the controls, but there was less difference between these two groups in using the “stir-frying” and “deep-frying” cooking methods (Table 2). This could be because stir-frying is the most common cooking method in Chinese-style cooking, and almost every participant in our study had used it quite frequently; similarly, the deep-frying method was less used by most of the participants. Accordingly, only the pan-frying method shows a significant association with lung cancer in our study (Table 5). Nevertheless, pan-frying, stir-frying, and deep-frying typically involve heating oil to a high temperature, resulting in a great amount of smoke. According to previous reports^39–42^, various kinds of mutagens and human carcinogens — such as benzo[a]pyrene, polycyclic aromatic hydrocarbons, and formaldehyde — could be released from oil into COFs. A previous report indicated that Chinese-style cooking in particular can result in especially high exposure to COF; it is likely that Chinese and other at-risk communities face similarly high levels of risk^17^.

With regard to types of cooking oils (vegetable oil and lard), more cases than controls used lard frequently (Table 2). Additionally, based on the results of Table 5, it may be concluded that exposure to COFs from lard increases the risk of lung cancer more than that from vegetable oil. This result is consistent with a previous report that use of vegetable oil (for example, palm oil) could reduce the production of aldehydes in COF exposure, especially for long-chain aldehydes such as hexanal and t,t-2,4-DDE^43^. However, only a few participants in our study frequently used lard in cooking, so a larger sample size would be required for confirmation.

The same analyses as in Tables 3–5 were also conducted on the cases of lung adenocarcinoma subtype. These results, shown in Supplementary Tables 2–4, were similar to those revealed in the main analyses.

Not only environmental factors but also genetic factors are thought to be implicated in the mechanism underlying the pathogenesis of lung cancer in nonsmokers. In the past few years, several studies investigated not only the associations between genetic polymorphisms and lung cancer risk but the interactions between these polymorphisms and COF exposure on susceptibility to lung cancer^44–50^. Although some of them were detected to be significant, there are many remaining uncertainties. Further studies will be conducted to clarify the gene–environment interactions and may provide insights into lung cancer in nonsmokers.

Our results should be interpreted with some caution. Apart from COF exposure, cooking time-years could be a surrogate for other exposures or factors, such as occupational exposure. In this case, COF exposure could be confounded by certain factors when using cooking time-years. For example, cooking time-years could be related to a full-time homemaker, and thus reflect some other risk factors such as exposure to indoor radon and/or lack of exposure to outdoor air pollution. Indeed, the positive correlation between cooking time-years and being a homemaker was observed in our data, with a correlation coefficient of 0.15 (*P* = 5.25e-14). However, different from cooking time-years, being a homemaker was negatively associated with lung cancer risk (see Supplementary Table 5). Similarly, the fume extractor use ratio could also be a surrogate for other factors, such as socio-economic status. Limited by the availability of data, we could remove only part of the confounding effect by adjusting for education.

The potential for recall bias exists in our study, especially as the diagnoses were known to the lung cancer cases. This could lead to, for example, underreporting of fume extractor use in the case group. For the cooking time-years and the other self-reported possible confounders, recall bias may result in bias toward the null because the link between these variables and lung cancer was not commonly acknowledged^24^. Nevertheless, recall inaccuracies on cooking time-years were likely, resulting in misclassification. To examine the impact of misclassification of cooking time-years, we randomly selected 3%, 5%, and 10% of participants and artificially reclassified their categories of cooking time-years. Then we randomly reset the value of cooking time-years for each of the participants selected according to her reclassified category. A dose–response trend was also evident when 5% of participants were reclassified. (The median p-values of the dose–response trend for 100 repetitions were 0.041, 0.045, and 0.086, corresponding to the cases of 3%, 5%, and 10%, respectively).

The ratio of the case number in this study compared to the number of all recorded cases of female lung cancers in Taiwan during the study period is about 4.8%^51^. However, the possibility of selection and self-selection bias was considered low. Since most lung cancer patients were diagnosed and treated at university or general hospitals in Taiwan, the demographic and clinical characteristics of the patients in this study were compatible with those in Taiwan in general, and the case group could be reasonably representative of the lung cancer population of Taiwan. Also note that, in GELAC, all controls were randomly selected, with frequency matching to the cases by age, gender, and ethnic background. Participation rates among cases and controls were about 94% and 87%, respectively. There were substantial numbers of “unknown” responses for several variables, including lung cancer in first-degree relatives in the control group, fume extractor use ratio in both of case and control groups, and habits of using each cooking method in the case group. We treated “unknown” responses as missing data and excluded the individuals who had any missing value on the covariates considered in the model for each analysis. Hence the numbers of cases and controls included in the analyses were substantially less than the original numbers. This could also have raised concerns on the representativeness of the analysis results.

There were several other limitations to this study. First, inadequate control for confounding factors remains a concern due to a lack of or incomplete information on other important potential confounders, such as residential radon exposure, other indoor combustions (e.g., incense burning, mosquito coils), outdoor air pollution, dietary factors, and intensity and duration of SHS exposure. We did not collect further information with respect to cooking behavior, such as the number of dishes per meal and cooking habits for other methods. Therefore, we could not quantitatively estimate and compare the relative fractions of the COF exposure resulting from different cooking methods using a joint model. In addition, we had no information on the types of vegetable oil used in cooking — such as soybean, olive, peanut, palm, and rapeseed — and therefore did not evaluate the association between lung cancer risk and exposure to COFs from different types of vegetable oils. We also did not evaluate the association between lung cancer risk and COF exposure from different cooking methods depending on vegetable oil or lard, because our questionnaires were unable to clarify, for example, whether a participant used lard only while deep-frying.

In conclusion, this study provides for future research the use of an objective method to measure lifetime exposure to COFs (cooking time-years), which not only gives strong evidence that cumulative exposure to COFs increases the risk of lung cancer but also indicates that long-term use of a fume extractor could greatly reduce this risk. For public health impact, this study suggests that kitchens be equipped with a fume extractor and that this device be used. Furthermore, cooking habits should be changed by increasing steaming and poaching food instead of pan-frying it, and by using vegetable oil rather than lard, which might reduce the effect of exposure to COFs and in turn reduce the lung cancer risk for non-smoking Han Chinese women.

## Supplementary information

Supplementary information.

## Data Availability

Because the participants did not give explicit written consent that their data can be made publicly available, data will not be shared.
