# Supplementary material for: Impact of cooking oil fume exposure and fume extractor use on lung cancer risk in non-smoking Han Chinese women
Source: Sci Rep. 2020 Apr 21;10:6774. doi: 10.1038/s41598-020-63656-7 (PMC7174336; doi:10.1038/s41598-020-63656-7)
Supplement: Supplementary file 1 — Supplementary information. [file 41598_2020_63656_MOESM1_ESM.pdf]

Supplementary Information

Impact of cooking oil fume exposure and fume extractor use on lung cancer risk in non-smoking Han Chinese women

Tzu-Yu Chen[1+], Yao-Hwei Fang[1+], Hui-Ling Chen[1], Chin-Hao Chang[1,2], Hsin Huang[1,3], Yi-Song Chen[1,4], Kuo-Meng Liao[5], Hsiao-Yu Wu[1], Gee-Chen Chang[6,7], Ying-Huang Tsai[8], Chih-Liang Wang[9], Yuh-Min Chen[10,11], Ming-Shyan Huang[12], Wu-Chou Su[13], Pan-Chyr Yang[14], Chien-Jen Chen[15], Chin-Fu Hsiao[1]* and Chao A. Hsiung[1]*

Affiliations:

[1]Institute of Population Health Sciences, National Health Research Institutes, Zhunan, Taiwan

[2]Department of Medical Research, National Taiwan University Hospital, Taipei, Taiwan

[3]Department of Nursing, Fu Jen Catholic University, Taipei, Taiwan

[4]Department of Microbiology, National Taiwan University College of Medicine, Taipei, Taiwan

[5]Division of Endocrinology & Metabolism, Taipei City Hospital, Zhongxiao Branch, Taipei, Taiwan

[6]Faculty of Medicine, School of Medicine, National Yang-Ming University, Taipei, Taiwan

[7]Division of Chest Medicine, Department of Internal Medicine, Taichung Veterans General Hospital, Taichung, Taiwan

[8]Department of Pulmonary and Critical Care Medicine, Chiayi Chang Gung Memorial Hospital, Chang Gung Medical Foundation, Chiayi, Taiwan

[9]Department of Thoracic Medicine, Chang Gung Memorial Hospital, Taoyuan, Taiwan

[10]School of Medicine, National Yang-Ming University, Taipei, Taiwan

[11]Department of Chest Medicine, Taipei Veterans General Hospital, Taipei, Taiwan

[12]Department of Internal Medicine, E-Da Cancer Hospital, School of Medicine, I-Shou University and Kaohsiung Medical University, Kaohsiung, Taiwan

[13]Department of Internal Medicine, National Cheng Kung University Hospital, College of Medicine, National Cheng Kung University, Tainan, Taiwan

[14]Department of Internal Medicine, National Taiwan University Hospital, Taipei, Taiwan

[15]Genomics Research Center, Academia Sinica, Taipei, Taiwan

+These authors contributed equally to this work and are considered co-first authors.

*Correspondence to: Chin-Fu Hsiao and Chao A. Hsiung,

Supplementary Table 1. Odds ratios and 95% confidence intervals for cooking time-years and risk of lung cancer between cases and controls regarding to different fume extractor use ratio in the Taiwan GELAC study during 2002–2010

	**Short-term use of fume extractor**				**Long-term use of fume extractor**			
	**Controls (389)**	**Cases (250)**			**Controls (522)**	**Cases (653)**		
**Cooking time-years**	**N (%)**	**N (%)**	**aOR**[a]	**95% CI**	**N (%)**	**N (%)**	**aOR**	**95% CI**
≤10	148 (38.0)	143 (57.2)	1		148 (28.4)	143 (21.9)	1	
11–110	226 (58.1)	59 (23.6)	3.50	1.74, 7.30[*b]	333 (63.8)	451 (69.1)	1.53	1.13, 2.08[*]
>110	15 (3.9)	48 (19.2)	6.27	2.52, 16.32[*]	41 (7.8)	59 (9.0)	2.29	1.14, 4.70[*]

Abbreviations: aOR, adjusted odds ratio; OR, odds ratio; CI, confidence interval.

[a]aOR: The OR was adjusted for age, education, lung cancer in first-degree relatives, exposure to second-hand smoke at home and the workplace, exposure to second-hand smoke of relatives who smoked, history of hormone-replacement therapy, history of oral contraceptive use, homemaker status, and history of being a chef.

[b]* indicates that the 95% CI of the OR does not include 1.

Supplementary Table 2. Odds ratios and 95% confidence intervals for cooking time-years and risk of lung adenocarcinoma between cases and controls in the Taiwan GELAC study during 2002–2010

	**Crude model**				**Adjusted model**			
**Cooking time-years**	**Controls (1273)**	**Adenocarcinoma (1089)**			**Controls (787)**	**Adenocarcinoma (933)**		
	**N (%)**	**N (%)**	**OR**	**95% CI**	**N (%)**	**N (%)**	**aOR[a]**	**95% CI**
≤10	204 (16.0)	125 (11.5)	1		148 (18.8)	116 (12.5)	1	
11–60	378 (29.7)	368 (33.8)	1.59	1.22, 2.07*[b]	239 (30.4)	312 (33.5)	1.61	1.16, 2.23*
61–110	421 (33.1)	338 (31.0)	1.31	1.01, 1.71*	252 (32.0)	292 (31.4)	1.77	1.17, 2.67*
111–160	223 (17.5)	198 (18.2)	1.45	1.08, 1.95*	121 (15.4)	160 (17.2)	2.05	1.12, 3.81*
>160	47 (3.7)	60 (5.5)	2.08	1.34, 3.26*	27 (3.4)	51 (5.5)	3.12	1.28, 7.76*
								P_{trend} = 0.0366

Abbreviations: aOR, adjusted odds ratio; OR, odds ratio; CI, confidence interval.

[a]aOR: The OR was adjusted for age, education, lung cancer in first-degree relatives, exposure to second-hand smoke at home and the workplace, exposure to second-hand smoke of relatives who smoked, history of hormone-replacement therapy, history of oral contraceptive use, homemaker status, and history of being a chef.

[b]* indicates that the 95% CI of the OR does not include 1.

Supplementary Table 3. Adjusted odds ratios and 95% confidence intervals of fume extractor use ratio between lung adenocarcinoma cases and healthy controls in the Taiwan GELAC study during 2002–2010

	Controls	Adenocarcinoma		
	**N (%)**	**N (%)**	**aOR[a]**	**95% CI**
Overall (Cooking time-years >10)				
Short-term use (ratio 0–0.33)	41 (7.3)	89 (12.4)	1	
Medium-term use (ratio 0.34–0.66)	144 (25.8)	184 (25.7)	0.64	0.41, 1.00
Long-term use (ratio 0.67–1)	374 (66.9)	442 (61.9)	0.52	0.33, 0.80*[b]
Cooking time-years 11–110				
Short-term use (ratio 0–0.33)	26 (5.9)	53 (9.8)	1	
Medium-term use (ratio 0.34–0.66)	80 (18.2)	99 (18.2)	0.65	0.36, 1.16
Long-term use (ratio 0.67–1)	333 (75.9)	391 (72.0)	0.55	0.32, 0.93*
Cooking time-years >110				
Short-term use (ratio 0–0.33)	15 (12.5)	36 (20.9)	1	
Medium-term use (ratio 0.34–0.66)	64 (53.3)	85 (49.4)	0.72	0.34, 1.48
Long-term use (ratio 0.67–1)	41 (34.2)	51 (29.7)	0.46	0.19, 1.07

Abbreviations: aOR, adjusted odds ratio; OR, odds ratio; CI, confidence interval.

[a]aOR: The OR was adjusted for age, education, categories of cooking time-years, lung cancer in first-degree relatives, exposure to second-hand smoke at home and the workplace, exposure to second-hand smoke of relatives who smoked, history of hormone-replacement therapy, history of oral contraceptive use, homemaker status, and history of being a chef.

[b]* indicates that the 95% CI of the OR does not include 1.

Supplementary Table 4. Odds ratios and 95% confidence intervals for different cooking habits and risk of lung adenocarcinoma between cases and controls in the Taiwan GELAC study during 2002–2010

**Cooking habits**		**Controls** N (%)	**Adenocarcinoma** N (%)	**aOR**[b]	**95% CI**
Cooking methods[a]					
Pan-frying	Low frequency (CDW ≤ 5)	383 (57.8)	349 (44.6)	1	
	High frequency (CDW > 5)	280 (42.2)	433 (55.4)	1.55	1.24, 1.94*[c]
Stir-frying	Low frequency (CDW < 7)	224 (32.7)	222 (28.0)	1	
	High frequency (CDW = 7)	462 (67.3)	572 (72.0)	1.18	0.92, 1.51
Deep-frying	Low frequency (CDW = 0)	411 (60.1)	451 (56.9)	1	
	High frequency (CDW > 0)	273 (39.9)	342 (43.1)	1.09	0.88, 1.37
Cooking oils					
Vegetable oil frequently		374 (54.1)	439 (50.8)	1	
Vegetable oil and lard		305 (44.1)	396 (45.8)	1.00	0.79, 1.27
Lard frequently		15 (1.7)	30 (3.5)	1.49	0.77, 3.01

Abbreviations: aOR, adjusted odds ratio; CDW, weighted average of cooking days weekly; CI, confidence interval.

[a]The medians of CDW for pan-frying, stir-frying, and deep-frying are 5, 7, and 0, respectively.

[b]aOR: The OR was adjusted for age, education, categories of cooking time-years, lung cancer in first-degree relatives, exposure to second-hand smoke at home and the workplace, exposure to second-hand smoke of relatives who smoked, history of hormone-replacement therapy, history of oral contraceptive use, homemaker status, and history of being a chef.

[c]* indicates that the 95% CI of the OR does not include 1.

Supplementary Table 5. Odds ratios and 95% confidence intervals for each covariate and risk of lung cancer between cases and controls in the Taiwan GELAC study during 2002–2010

	**Univariate model**				**Multivariate model**			
	**Controls**	**Cases**			**Controls**	**Cases**		
**Covariate**	**N**	**N**	**OR**	**95% CI**	**N**	**N**	**OR**	**95% CI**
Age	1,302	1,302	1.00	0.99, 1.00	800	1,115	1.01	1.00, 1.02
Education	1,302	1,302	1.00	0.95, 1.06			0.97	0.90, 1.05
Lung cancer in first-degree relatives	915	1,242	2.16	1.55, 3.05*[a]			2.06	1.44, 3.01*
Second-hand smoke at home	1.268	1,276	1.43	1.22, 1.68*			1.34	1.10, 1.64*
Second-hand smoke at work	1,302	1,300	1.37	1.12, 1.68*			1.18	0.92, 1.52
Second-hand smoke of relatives	1,301	1,299	1.22	1.02, 1.47*			1.08	0.86, 1.34
History of hormone-replacement therapy	1,221	1,248	0.93	0.76, 1.14			0.75	0.59, 0.95*
History of oral contraceptive use	1,237	1,229	1.22	0.92, 1.62			0.98	0.71, 1.35
Homemaker status	1,283	1,297	0.64	0.48, 0.84*			0.61	0.44, 0.85*
History of being a chef	1,302	1,301	1.03	0.75, 1.41			1.04	0.70, 1.56

Abbreviations: OR, odds ratio; CI, confidence interval.
[a]* indicates that the 95% CI of the OR does not include 1.
